# Refinement of intermediate-risk Karyotypes according to the IPSS-R in patients with myelodysplastic neoplasms (MDS)

**DOI:** 10.1007/s00277-025-06443-6

**Published:** 2025-06-09

**Authors:** K. Nachtkamp, F. Schulz, A. Kasprzak, C. Strupp, B. Hildebrandt, M. Pfeilstöcker, P. Valent, B. Blum, A. Giagounidis, K. Götze, V. Flatten, S. Dietrich, G. Kobbe, D. Haase, N. Gattermann, U. Germing

**Affiliations:** 1 Department of Hematology, Oncology and Clinical Immunology, Heinrich Heine University, Duesseldorf, Germany; 2 Institute of Human Genetics, Heinrich Heine University, Duesseldorf, Germany; 3 Medical Department for Hematology and Oncology, Hanusch Hospital, Vienna, Austria; 4 Department of Internal Medicine I, Division of Hematology and Hemostaseology, Medical University of Vienna, Vienna, Austria; 5 Ludwig Boltzmann Institute for Hematology and Oncology, Medical University of Vienna, Vienna, Austria; 6 Service d’hématologie, Département d’oncologie, Centre Hospitalier Universitaire Vaudois, and Lausanne University (UNIL), Lausanne, Switzerland; 7 Department of Hematology, Oncology and Palliative Care, Marien Hospital, Duesseldorf, Germany; 8 Department of Hematology and Oncology, Klinikum Rechts der Isar, Technische Universität Muenchen, Munich, Germany; 9 Department of Hematology and Medical Oncology, Georg August University, Göttingen, Germany; 10 Department of Hematology, Oncology and Clinical Immunology, Heinrich-Heine-University, Moorenstr. 5, 40225 Düsseldorf, Germany

**Keywords:** IPSS-R, Prognosis, Intermediate-risk, New karyotype risk groups, Myelodysplastic neoplasms

## Abstract

MDS patients show a heterogenous prognosis which can be stratified by the IPSS-R in order to derive therapeutic implications. Based on 618 patients with myelodysplastic neoplasms belonging to the cytogenetic intermediate-risk group according to IPSS-R, we show that this group is heterogeneous in terms of overall survival and cumulative risk of AML. The group can be reorganized into subgroups according to their prognostic impact. A small subgroup of patients with isolated -X or der(1;7) can be regarded as very-low-risk patients with a median survival time of 112 months and a cumulative AML progression rate of 9% after 2 years. A larger group of patients with either diverse aberrations of one chromosome or -Y + one additional aberration shows a benign course of the disease with a median survival time of 46 months and a cumulative AML progression rate of 26% after 2 years. A very large group of patients presenting with either + 8, +19, i(17q), + 21, +mar, del(9q), + 8 plus one other aberration, or del(7q) have a poor prognosis with a median survival time of 26 months and a cumulative AML progression rate of 32% after 2 years. In a very small set of patients with trisomy 11 the course of disease was similar to very-high-risk patients with a median survival time of 17 months only and a cumulative AML progression rate of 100% after 2 years. These findings could lead to a refinement of prognostic scoring systems such as the IPSS-R and the IPSS-M.

## Introduction

Chromosomal findings play a major role in the assessment of prognosis in patients with myelodysplastic neoplasms (MDS). The International Prognostic Scoring System (IPSS) defined three groups of karyotype anomalies that are associated with significantly different prognoses [1]. Based on these findings, the WHO-adjusted IPSS (WPSS) and revised IPSS (IPSS-R) assigned greater weight to chromosomal aberrations, as compared to blast percentage and hematopoietic insufficiency [2, 3]. An even better utilization of chromosomal findings for MDS prognostication was provided by an international working group consisting of the German-Austrian MDS Study Group, the International MDS Risk Analysis Workshop, the Spanish Hematological Cytogenetics Working Group, and the International Working Group on MDS Cytogenetics [4]. This consortium, led by colleagues from Goettingen, defined five cytogenetic risk groups that differ significantly in terms of overall survival (OS) and cumulative AML probability (Table 1).

**Table 1:** Chromosomal categories as defined by Schanz et al. [4] and prognosis according to IPSS-R original data [3] as well as in the present cohort (*n* = 3207)

Risk category	Karyotype groups	Percentage of patients [3]	Median survival, (months) [3]	Percentage of patients (present cohort)	Median survival (months) in the present cohort
Very low	del(11q), -Y	4%	65	4%	98
Low	normal, del(5q), del(12p), del(20q), del(5q), double anomaly including del(5q)	72%	58	58.4%	64
Intermediate	+ 8, del(7q), i(17q), + 19, any other single or double anomaly, independent clones	13%	32	17.7%	36
High	−7, inv(3)/t(3q)/del(3q), 2 anomalies including − 7,/del7q, complex with 3 anomalies	4%	18	8.2%	24
Very high	complex with > 3 anomalies	7%	8.5	11.7%	11

The proportion of karyotypes with unclear significance was minimized by collecting a large data set from many centers. The resulting cytogenetic categorization was subsequently integrated into the revised version of the IPSS [3], and together with molecular studies, has since been used a as a basis for the development of the molecular IPSS (IPSS-m) [5]. The IPSS-R has proven to be very robust and useful, both in clinical trials and daily clinical decision making [6, 7].

The cytogenetic intermediate-risk group according to IPSS-R features aberrations that have a significantly worse prognostic impact than good-risk karyotypes, while imparting a better prognosis than high-risk karyotypes such as −7 [1, 3]. Nevertheless, these intermediate-risk karyotypes represent a heterogeneous group, partly since many single and double aberrations were put in the intermediate-risk group simply due to their rarity so they could not be safely assigned to the low-risk or high-risk categories. As a result, this group merges patients with a more favorable prognosis as well as patients with a considerably higher risk for AML development. A better prognostic characterization of those relatively rare cytogenetic aberrations not adequately addressed in the IPSS-R may help to improve stratification of this group. We therefore examined a large group of patients with chromosomal findings belonging to the intermediate-risk group as defined by Schanz et al. [4].

## Methods

We analyzed 618 patients from the German-Austrian-Swiss MDS group with intermediate-risk karyotype, as defined in the IPSS-R, with regard to OS and risk of AML progression. Patients were assessed for survival from diagnosis to time of last follow up/death (data lock December, 31 th, 2023), as well as for progression to AML. 63% of the patients died in the observation period. Median observation time was 21 months (1–367). We analyzed each karyotype aberration separately as a first step. Aberrations with a similar prognosis were grouped together, but collections of < 10 patients were not regarded large enough to constitute a separate prognostic group. A minimum of 10 metaphases were analyzed [8, 9]. We estimated survival probability and cumulative risk of AML evolution using the Kaplan-Meier method.

## Results

Different categories of chromosomal findings within cytogenetically intermediate-risk patients according to the IPSS-R are listed in Table 2.

**Table 2:** Frequency of chromosomal findings within intermediate risk patients by cytogenetics and prognostic meaning

Types of chromosomal aberrations	frequency (%)	median survival (months)	cum. AML evolution after 2 and 5 years	
+1/der(1;7)	18 (2.4)	90	0%	0%
-X	15 (2.4)	n.r.	8%	23%
-Y + one additional aberration	14 (2.3)	58	16%	16%
diverse aberrations of one chromosome	197 (31.8)	40	23%	23%
del(7q)	30 (4.9)	38	28%	50%
+21	10 (1.6)	32	48%	48%
diverse aberrations of two chromosomes	90 (14.4)	31	26%	52%
del(9q)	11 (1.8)	26	31%	46%
+19	11 (1.8)	41	16%	16%
+8	146 (23.7)	23	30%	54%
i(17q)	6 (1)	21	0%	0%
+mar	19 (3.1)	18	56%	56%
+8 + one additional aberration	41 (6.7)	27	38%	38%
+11	10 (1.6)	17	100%	100%
all patients	618 (100)	31	28%	39%

There are relatively small groups characterized by a specific single aberration with or without one additional aberration. The largest group of this type featured trisomy 8 (23.7%) and trisomy 8 with one additional aberration (6.7%). Altogether, about half of the patients showed a single aberration of any kind (31.8%) or a double aberration (14.4%), leading to a multitude of small, genetically distinct subgroups.

Table 2 also shows the strongly divergent survival times and cumulative risks of AML evolution after 2 and 5 years for the different groups of cytogenetic aberrations. The median OS of the entire group was 31 months. Patients with either unbalanced translocation (1;7) (der(1;7)) or -X had the longest median survival time (*n* = 33), and patients with -Y plus one additional aberration also had a favorable prognosis (*n* = 14).

Within the group of patients with any aberration of either chromosome 1, del(7q), + 21, or diverse aberrations of two chromosomes, median OS was about 3 years. Patients with either del(9q), + 19, +8, + 8 with one other aberration, or i(17q), had a median OS of about 2 years, and patients with either a marker chromosome or + 11 showed a median OS of about 1 and 0.5 years, respectively.

We stratified the cohort into four groups with significantly different median overall survival time and risk of AML evolution (*p* < 0.0005, Table 3).

**Table 3:** Prognostic impact of groups of aberrations (overall survival)

	Groups of aberrations	Frequency (%)	Median survival (months)	*p*	95%-CI	Number of events
1: Low	der(1;7); -X	33 (5.3)	112	<0.001	39.95–184.05	11 (33.3%)
2: Intermediate	diverse aberrations of 1 chromosome; -Y + 1 other aberration	211 (34.2)	46		32.95–59.04	124 (20.1%)
3: High	+ 8; +19; i(17q); +21, +mar; del(9q); +8 + 1 other aberration, del(7q), any 2 aberrations	364 (58.9)	26		21.95–30.05	118 (32.5%)
4: Very high	+ 11	10 (1.6)	17		8.32–25.68	8 (80%)

**Fig. 1:**
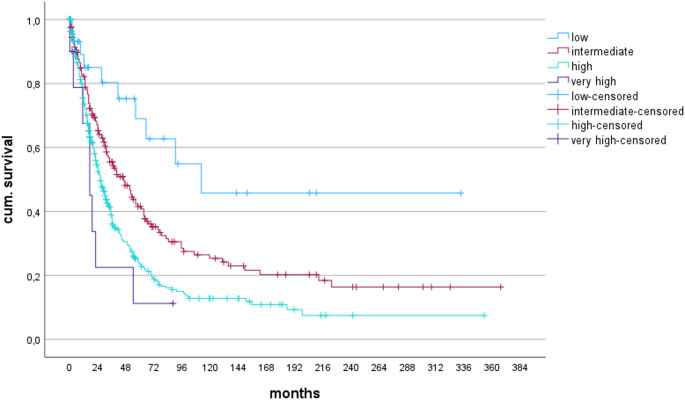
cumulative risk of survival according to the new karyotype risk categories (*p* < 0.0001)

Patients with + 11 (group 4, “very high”) showed the worst OS (17 months). OS was 26 months in the group of patients with either + 8, +19, i(17q), +mar, del(9q), + 21, +8 with 1 additional aberration, any aberration of 2 chromosomes, or patients with del(7q) (group 3, “high”). Patients with any single aberration or –Y with 1 additional aberration (group 2, “intermediate”) had a median survival time of 46 months, and patients with either der(1;7) or –X (group 1, “low”) had a median survival time of 112 months (Fig. 1/Table 4).

**Table 4:** Numbers at risk regarding OS

	24	48	72	96	120	144	168	192	216	240	264	288	312	336	360	months
Low	18	10	9	7	5	4	3	2	1	1	1	1	1	1		
Int.	114	68	36	28	23	18	15	13	10	8	6	4	2	1	1	
High	154	71	33	27	18	14	10	5	3	1	1	1	1	1		
Very high	2	2	1	0												

**Table 5:** Prognostic impact of groups of aberrations (cumulative risk of AML evolution)

	Groups of aberrations	AML after 2 years %	AML after 5 years %	*p*	Number of events
1: Low	der (1;7); -X	8.7	8*.7*	<0.001	2 (6.3%)
2: Intermediate	diverse aberrations of 1 chromosome; -Y + 1 other aberration	25.5	31.5		59 (28%)
3: High	+ 8; +19; i (17q); +21, +mar; del(9q); +8 + 1 other aberration, del(7q), any 2 aberrations	31.9	47.3		117 (32.2%)
4: Very high	+ 11	100	100		7 (70%)

**Fig. 2:**
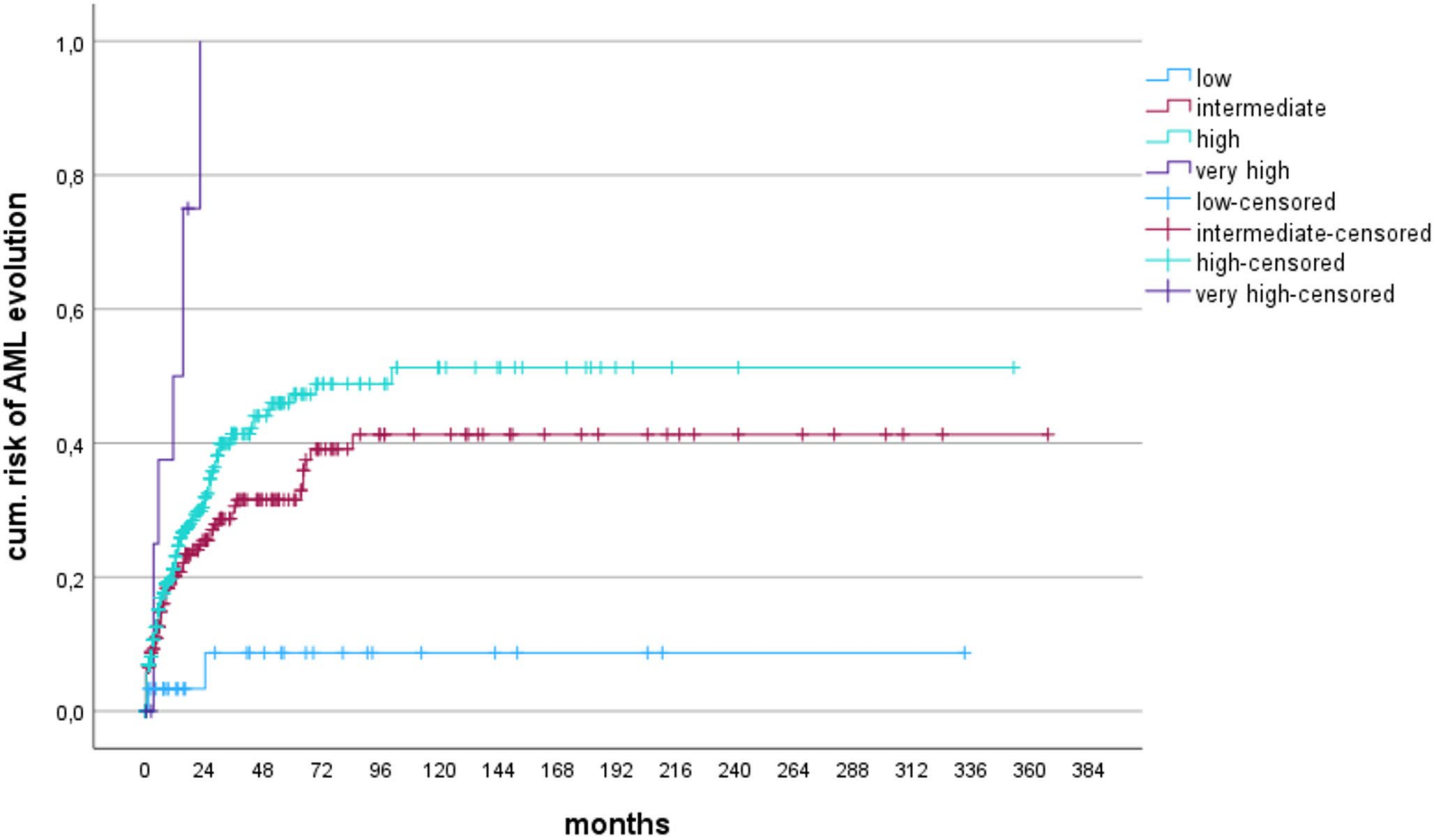
cumulative risk of AML evolution according to the new karyotype risk categories (*p* < 0.0001)

**Table 6:** Numbers at risk regarding AML evolution. The median numbers of clinical parameters bearing prognostic relevance and utilized within the IPSS-R and their distribution within the newly formed chromosomal risk groups are shown in Table 7

	24	48	72	96	120	144	168	192	216	240	264	288	312	336	360	months
**Low**	17	13	9	6	5	4	3	3	1	1	1	1	1	1		
**Int.**	100	60	31	24	21	16	14	11	9	7	6	4	2	1	1	
**High**	133	59	27	23	15	11	9	4	2	2	1	1	1	1		
**Very high**	0															

**Table 7:** Selected clinical parameters within regrouped intermediate risk cytogenetic cohort. We then analyzed the distribution of our regrouped chromosomal categories belonging to the cytogenetic intermediate-risk group by IPSS-R within the IPSS-R overall risk subgroups as shown in Table 8

		ANC	Hemoglobin level	Platelet count	Median BM-blast proportion
Regrouped intermediate risk cytogenetic	**Low**	1980 (168–6700)	9.5 (6–14)	83 (6–547)	4 (1–19)
	**Intermediate**	1910 (70–8900)	9.9 (5.1–15.3)	100 (7–591)	6 (0–19)
	**High**	1700 (0–9700)	9.3 (6.2–16.6)	109 (1–780)	4 (0–19)
	**Very high**	650 (150–6300)	9.3 (7.3–10.2)	52 (12–241)	18 (3–19)

**Table 8:** Crosstab of regrouped intermediate risk cytogenetic cohort and IPSS-R risk groups

			IPSS-R				total
			low	intermediate	high	very high	
Regrouped intermediate risk cytogenetic	**low**	n	11	11	7	3	32
		%	34.4%	34.4%	21.9%	9.4%	100%
	**intermediate**	n	52	54	70	36	212
		%	24.5%	25.5%	33%	17%	100%
	**high**	n	67	114	119	64	364
		%	18.4%	31.3%	32.7%	17.6%	100%
	**very high**	n	0	1	3	6	10
		%	0%	10%	30%	60%	100%
total		n	130	180	199	109	618
		%	21%	29.1%	32.2%	17.6%	100%

Correspondingly, 100% of patients with + 11 (group 4) developed AML within 5 years (*n* = 7), whereas the risk of AML was significantly lower in groups 2–3, and less than 10% in group 1 (Tables 5; Fig. 2/Table 6)

It becomes evident that 66% of low-risk cytogenetic patients with a previously demonstrated median overall survival time of 112 months are categorized into IPSS-R intermediate, high or very high groups. Likewise, 50% of cytogenetically regrouped intermediate risk patients with a median survival time of 46 months are IPSS-R high or very high. When assessing high-risk subgroups, high-risk patients by our newly formed subgroup with a median overall survival time of 26 months were diametrically categorized as low or very high by IPSS-R in 18% of cases each. Lastly, 40% of very high-risk patients by our stratification methods with a median overall survival of 17 months are stratified into intermediate or high-risk by the IPSS-R. Finally, we assessed if the restratification of patients leads to a better discrimination of the risk groups of the IPSS-R. The restratified IPSS-R has a logrank value of 44.5 as compared to 26.7 of the original IPSS-R, the Breslow test was 56.6 vs. 29.9 and the Tarone test was 54.7 vs. 26.7, which indicates a somewhat better distribution of the patients.

## Discussion

Based on 618 patients with myelodysplastic neoplasms belonging to the cytogenetic intermediate risk group according to IPSS-R, we demonstrate that this group is more heterogeneous in terms of prognosis than usually assumed, regarding both overall survival and cumulative risk of AML. Other authors have tried identifying additional parameters to further prognosticate intermediate risk patients such as Benton et al. To our knowledge, these focused on clinical/laboratory parameters and did not address cytogenetics, though [10].

By reorganizing the cytogenetic findings according to their prognostic impact, we were able to define four distinct subgroups within the cytogenetic intermediate-risk group according to IPSS-R. A very small group of patients with isolated -X or der(1;7) are comparable to “very-low-risk” MDS patients. A larger group of patients with diverse aberrations of one chromosome or with -Y in combination with one other aberration showed a relatively benign course of the disease. The largest group of patients with either + 8, +19, i(17q), + 21, +mar, del(9q), + 8 plus one other aberration or del(7q) had a worse prognosis. In a small set of patients with trisomy 11, the course of disease was strikingly similar to “very-high-risk” patients, with an extremely high risk of progressing to AML. We are aware of the limitation that patient numbers within the low and very-high-risk groups are low, supporting our notion to have our findings validated by other cohorts.

We tried to shed light on the potential influence of parameters that are part of the categorization into the IPSS-R on the newly formed cytogenetic subgroups. We separately analyzed our newly established low-risk and intermediate-risk groups, then the intermediate-risk and high-risk groups, as well as the high-risk and very-high-risk subgroups with regards to statistically significant differences concerning hemoglobin levels, neutrophil counts, platelet counts and medullary blast count as central parameters dictating stratification into IPSS-R risk subgroups. A systematic difference could only be shown between the very-high-risk subgroup and the high-risk group and within this analysis exclusively with regards to the medullary blast count (mean blast count 8.3% in the high-risk group versus 16.1% in the very high-risk group, t-test: one-tailed *p* = 0.02). The results corroborate the independent prognostic effect of the refined subgroups established by our analyses on OS and risk for AML evolution.

We also considered looking into the potential influence of therapeutic measures on the cohort. As shown in Table 9, the majority of the overall cohort had received best supportive care only (73%).

**Table 9:** Therapeutic interventions within regrouped intermediate-risk cytogenetic cohort

			BSC	HMA	induction chemo	allogeneic PBSCT	total
Regrouped intermediate risk cytogenetic	**low**	n	24	2	1	6	33
		%	72.7%	6.1%	3.0%	18.2%	100%
	**intermediate**	n	157	18	25	11	211
		%	74.4%	8.5%	11.8%	5.2%	100%
	**high**	n	263	36	54	11	364
		%	72.3%	9.9%	14.8%	3%	100%
	**very high**	N	7	0	2	1	10
		%	70.0%	0%	20.0%	10%	100%
total		n	451	56	82	29	618
		%	73.0%	9.1%	13.3%	4.7%	100%

9.1% of patients received hypomethylating agents (HMA), 13.3% received induction chemotherapy and an allogeneic stem cell transplantation was performed in 4.7% of cases. Within our refined subgroups, no statistically significant preponderance of any therapeutic intervention was demonstrable. This may be in line with the aforementioned finding that our newly formed subgroups of the cohort of intermediate-risk patients by the cytogenetic IPSS-R do not well overlap with the IPSS-R risk subgroups. Indeed, when separating the overall group by the IPSS-R and correlating these subgroups with therapeutic measures, a clear association with therapeutic intervention beyond BSC and higher-risk subgroups becomes evident as the therapeutic support by BSC drops from 87.8% in IPSS-R low-risk patients to 56% in IPSS-R very-high-risk patients in the respective cohort (*p* < 0.001). This in our opinion strengthens the independent effect of cytogenetic subgroups as defined by our analyses on the prognosis of intermediate-risk MDS patients according to cytogenetic IPSS-R categorization.

**Fig. 3:**
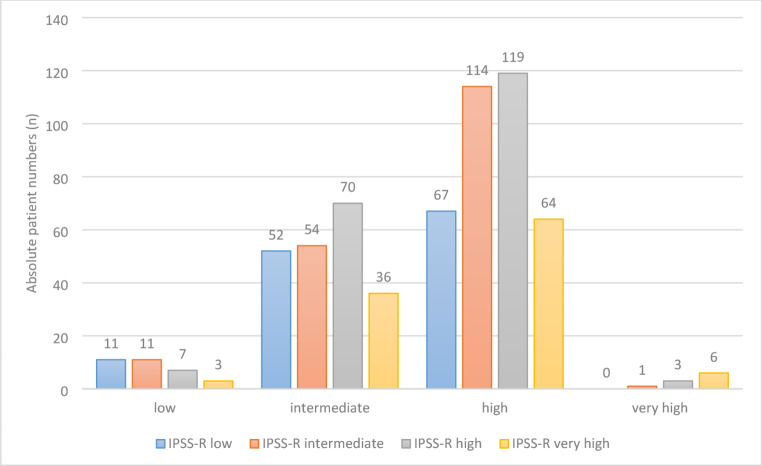
Distribution of regrouped cytogenetic intermediate risk patients within IPSS-R risk groups

Our data could demonstrate that a substantial proportion of patients clearly demonstrating an either better or worse prognosis by cytogenetic regrouping than suggested by their original classification as cytogenetically intermediate-risk are categorized into IPSS-R groups with an either better or worse median overall survival time and risk for AML evolution (Fig. 3).

There was no patient falling into the IPSS-R very-low-risk category which is logical as two points are attributed to cytogenetically intermediate-risk patients and very-low-risk IPSS-R require ≤ 1.5 points maximum. In total, 262 patients (42% of the cohort of intermediate-risk cytogenetics) would need to be regrouped into a different IPSS-R category when acknowledging the results of these analyses. It appears that at least two of the subgroups, namely the one with a very good prognosis on the one hand, and the one with a very poor prognosis on the other hand, should not belong to an intermediate risk group. Since the two larger subgroups also differ significantly in terms of OS and risk of AML evolution, the question arises if the entire cytogenetic intermediate-risk group according to IPSS-R is dispensable and patients should be assigned to one of the other cytogenetic risk groups in the IPSS-R. Before proposing such a change, which may foster more accurate risk assessment using the IPSS-R, validation of our new cytogenetic risk categorization should be obtained using an independent data set, especially in the light of small patient numbers in parts of the subgroups. It may also be of interest to investigate whether the prognostic impact of the different types of karyotype anomalies that we identified within the cytogenetic intermediate-risk group may be paralleled by differential effects on clonal expansion and evolution or the choice of treatment [11, 12]. For example, the high AML progression rate in patients with acquired trisomy 11 and the very low AML progression rate of der(1;7) and -X may be associated with corresponding paces of chromosomal and molecular genetic evolution or a difference in therapeutic attitude (best supportive care versus intensive treatment with allogeneic stem cell transplantation). Our data leads us to propose classifying our low-risk and very-high-risk patients according to cytogenetics into “high risk-like” and “low risk-like” subgroups for therapeutic decision-making. Within the cohort used for these analyses there was only limited availability of information on NGS data, unfortunately. A next step could be correlating the findings of this study with NGS data in a validation cohort for potential integration of the new cytogenetic risk groups into the IPSS-M.

## Data Availability

Original data sharing not possible due to SOP of MDS Registry. However, pseudonymized data can be sent to other researchers on demand. The raw data supporting the conclusions of this article will be made available by the authors, without undue reservation.
